# Post-COVID Myoclonus-Ataxia Syndrome: A Case Report of Successful Recovery With Intravenous Immunoglobulin (IVIG) Treatment

**DOI:** 10.7759/cureus.96796

**Published:** 2025-11-13

**Authors:** Maryam Babar, Aymen Arain, Ali Hamide, Razi Rashid

**Affiliations:** 1 College of Medicine, Edward Via College of Osteopathic Medicine, Monroe, USA; 2 Neurology, University of Texas Medical Branch, Houston, USA

**Keywords:** covid-19, gait ataxia, healthcare access barriers, myoclonus, neurological symptoms, nystagmus

## Abstract

The SARS-CoV-2 virus responsible for COVID-19 can trigger multisystemic syndromes that persist beyond the acute infection. Among these, neurological complications have become increasingly recognized. Reported outcomes include headaches, encephalitis, encephalopathy, anosmia, ageusia, Guillain-Barré syndrome, and skeletal muscle involvement such as myalgia and myasthenia gravis. Myoclonus-ataxia syndrome has emerged as a rare post-infectious complication. We describe an immunocompetent adult who developed post-COVID-19 myoclonus-ataxia with a distinct post-infectious timeline. The patient had minimal response to corticosteroids but experienced rapid and marked recovery with intravenous immunoglobulin (IVIG). Delayed access to IVIG due to insurance limitations prolonged disease progression. This case underscores the importance of individualized treatment strategies in post-COVID neurological syndromes and highlights the impact of systemic healthcare barriers. Given the rarity of this presentation and the variable treatment responses observed, this report contributes to the understanding of post-COVID neurological complications and their management.

## Introduction

The coronavirus disease 2019 (COVID-19) pandemic has been associated with a wide range of neurological manifestations, ranging from anosmia and encephalopathy to Guillain-Barré syndrome. Recently, post-infectious myoclonus-ataxia has been described as a rare but disabling complication. Post-infectious neurological syndromes are thought to occur when the immune system remains dysregulated after a viral illness, leading to autoimmune-mediated inflammation of neural pathways. Although corticosteroids are often used as first-line therapy, responses can be variable, and there is limited guidance on second-line interventions [1]. Here, we report a case of post-COVID myoclonus-ataxia in an immunocompetent adult who experienced minimal response to corticosteroids but showed rapid and significant recovery following intravenous immunoglobulin (IVIG) therapy. This report describes a rare case of steroid-resistant post-COVID myoclonus-ataxia that improved with IVIG, adding to emerging evidence of immune-mediated neurological complications following SARS-CoV-2 infection. This case highlights both the therapeutic potential of IVIG and the barriers to timely care created by insurance-related delays.

## Case presentation

A 56-year-old woman presented to the outpatient clinic with persistent imbalance, ataxic gait, brisk reflexes, and involuntary muscle jerks, following complete recovery from a prior SARS-CoV-2 infection. The patient also reported right-sided visual disturbance associated with nystagmus. Comorbidities included type II diabetes mellitus and hypertension, both of which were well-controlled during evaluation. The patient's vaccination status against SARS-CoV-2 at the time of infection is unknown.

Neurological physical exam revealed a wide-based ataxic gait, bilateral upper and lower limb myoclonus, and brisk deep tendon reflexes. Right-sided horizontal nystagmus was observed. The patient's functional status progressively declined over time, with frequent falls, worsening coordination, declining neuropsychiatric status with associated anxiety and depression, worsening memory, and increasing difficulty performing activities of daily living. 

Laboratory investigations, including a complete blood count, comprehensive metabolic panel, thyroid panel, creatine kinase isoenzyme panel, inflammatory markers, prolactin, and immunoglobulin levels, were largely within normal limits. Values can be observed in Tables 1-3. 

**Table 1:** Complete blood count labs. MPV: mean platelet volume; MCV: mean corpuscular volume; MCH: mean corpuscular hemoglobin; MCHC: mean corpuscular hemoglobin concentration; RDW: red cell distribution width.

Lab item	Value	Reference range	Units
Neutrophils	54.6	-	%
Absolute neutrophils	3385	1500-7800	cells/µL
Lymphocytes	36.9	-	%
Absolute lymphocytes	2288	850-3900	cells/µL
Monocytes	4.9	-	%
Absolute monocytes	304	200-950	cells/µL
Eosinophils	3.1	-	%
Absolute eosinophils	192	15-500	cells/µL
Basophils	0.5	-	%
Absolute basophils	31	0-200	cells/µL
MPV	10.9	7.5-12.5	fL
White blood cell count	6.2	3.8-10.8	Thousand/µL
Red blood cell count	5.01	3.80-5.10	Million/µL
Hemoglobin	15	11.7-15.5	g/dL
Hematocrit	44	35.0-45.0	%
MCV	87.8	80.0-100.0	fL
MCH	29.9	27.0-33.0	pg
MCHC	34.1	32.0-36.0	g/dL
RDW	13.4	11.0-15.0	%
Platelet count	226	140-400	Thousand/µL

**Table 2:** Complete metabolic panel labs. ALT: alanine aminotransferase; AST: aspartate aminotransferase; eGFR: estimated glomerular filtration rate; BUN: blood urea nitrogen; OR: odds ratio.

Lab item	Value	Reference range	Units
Protein, total	6.9	6.1-8.1	g/dL
Albumin	4.3	3.6-5.1	g/dL
Globulin	2.6	1.9-3.7	g/dL
Albumin/globulin ratio	1.7	1.0-2.5	(calc)
Bilirubin, total	0.6	0.2-1.2	mg/dL
Alkaline phosphatase	126	37-153	U/L
AST	20	10-35	U/L
ALT	22	6-29	U/L
Glucose	123	65-139	mg/dL
Urea nitrogen (BUN)	11	7-25	mg/dL
creatinine	0.68	0.50-1.05	mg/dL
eGFR non-African American	98	>OR = 60	mL/min/1.73 m²
eGFR African American	113	>OR = 60	mL/min/1.73 m²
BUN/creatinine ratio	Not applicable	6-22	(calc)
Sodium	139	135-146	mmol/L
Potassium	4.2	3.5-5.3	mmol/L
Chloride	105	98-110	mmol/L
Carbon dioxide	29	20-32	mmol/L
Calcium	9.5	8.6-10.4	mg/dL

**Table 3:** Various labs, including sedimentation rate, immunoglobulin A, thyroid panel, creatine kinase isoenzyme panel, and prolactin. CK-MB: creatine kinase-myocardial band isoenzyme; CK-MM: creatine kinase-muscle type isoenzyme; CK-BB: creatine kinase-brain type isoenzyme; T3: triiodothyronine; T4: thyroxine; T7 (free T4 index): free thyroxine index; Sed rate: erythrocyte sedimentation rate; OR: odds ratio.

Lab item	Value	Reference range	Units
Sed rate by modified Westergren	4	mm/h
Immunoglobulin A	327 H	47-310	mg/dL
T3 Uptake	30	22-35	%
T4 (thyroxine), total	9.7	5.1-11.9	mcg/dL
Free T4 index (T7)	2.9	1.4-3.8	-
CK-BB	None detected	None detected	-% of total
CK-MB	0	<5	-% of total
CK-MM	100	95-100	-% of total
Creatine kinase, total	59	29-143	U/L
Prolactin	3.1	-	ng/mL

MRI of the brain and cervical spine with and without contrast revealed no structural abnormalities; imaging files were unavailable for review, and only the official radiology report was accessible.

Initial treatment with a prednisone taper (50 mg daily for five days, followed by tapering) provided limited symptomatic benefit. Insurance-related delays postponed initiation of intravenous immunoglobulin (IVIG) therapy for nearly three years, during which her symptoms progressively worsened. Upon eventual initiation of IVIG, the patient demonstrated rapid and marked clinical improvement in myoclonus, gait stability, and cognitive clarity within several days of treatment. Supportive physical therapy was also continued to aid functional recovery.

Based on the temporal association with prior COVID-19 infection, the absence of structural abnormalities, and the clinical syndrome of ataxia with myoclonus, a post-infectious immune-mediated process was suspected.

## Discussion

**Figure 1:**
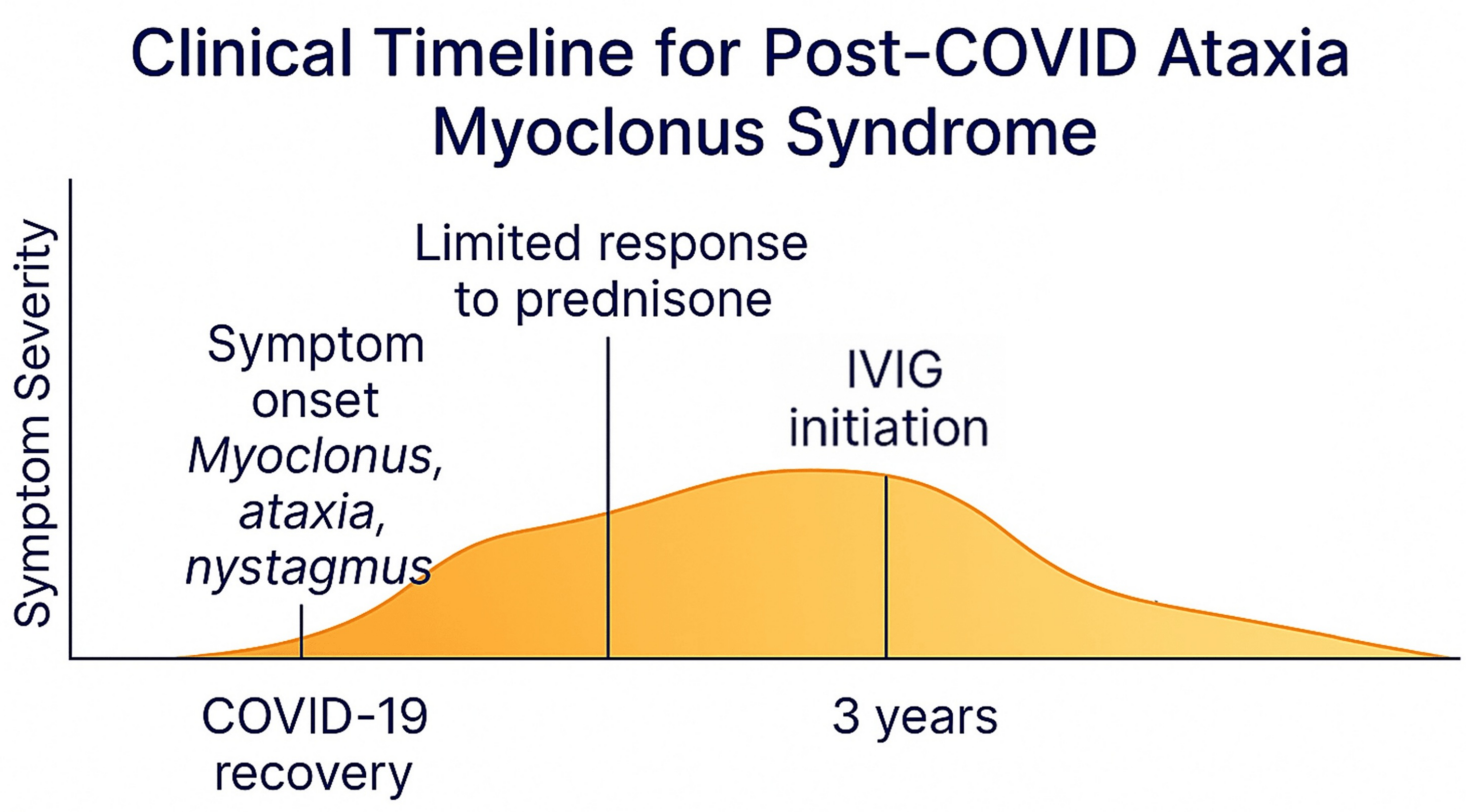
Clinical timeline for post-COVID myoclonus-ataxia Key takeaway: symptoms progressed over nearly three years without IVIG due to insurance delays; rapid improvement occurred shortly after IVIG initiation. IVIG: intravenous immunoglobulin.

This case describes a rare instance of myoclonus-ataxia following COVID-19 infection in an immunocompetent adult, with delayed but ultimately successful treatment with IVIG, as shown in Figure 1. Few cases have documented post-COVID myoclonus-ataxia. Most respond to steroids, but in this case, IVIG was required, suggesting a different immunopathology.

The partial effectiveness of glucocorticoids in this case suggests that ongoing inflammation or immune dysregulation contributed to the patient's symptoms. Glucocorticoids reduce neuroinflammation by suppressing pro-inflammatory cytokines, such as interleukin-6 (IL-6) and tumor necrosis factor-alpha (TNF-α), inhibiting leukocyte trafficking, and promoting anti-inflammatory signaling. However, in this case, a brief prednisone course may have been insufficient to reverse persistent antibody-mediated injury or immune dysregulation. The incomplete resolution of symptoms with steroid therapy indicates that other pathological mechanisms, such as autoantibody-mediated injury, might also be involved. In contrast, the patient experienced rapid improvement with IVIG, which exerts therapeutic effects by neutralizing residual viral antigens and modulating immune dysregulation. IVIG suppresses pro-inflammatory cytokines, blocks Fc receptor-mediated activation, and modulates B- and T-cell activity, addressing autoimmune or post-infectious pathways [1,2]. The limited response to corticosteroids and the rapid recovery with IVIG suggest an antibody- or immune-complex-mediated process affecting cerebellar and brainstem circuits. This outcome supports using IVIG in select post-COVID patients with worsening symptoms unresponsive to first-line anti-inflammatory treatment. While some reports recommend corticosteroids as the first line due to lower cost and wider availability, this case highlights their limitations [3]. For patients with prolonged, functionally limiting symptoms or poor steroid response, early IVIG may be justified and necessary.

Emerging evidence suggests that neurological dysfunction following cortical stressors may be partly mediated by epigenetic remodeling. In a rat model of cortical spreading depression, Passaro et al. demonstrated significant alterations in histone methylation patterns, including reduced H3K4 mono- and di-methylation, increased H3K9 di-methylation, and reduced levels of the histone methyltransferases mixed-lineage leukemia (MLL) and SET7. These changes suggest a shift toward a more transcriptionally repressive chromatin state [4]. These findings offer a mechanistic explanation for how neuronal insults can cause heritable yet reversible transcriptional changes, affecting excitability and plasticity long after the initial event. Similarly, the persistence of myoclonus and ataxia after a viral infection may reflect maladaptive epigenetic imprinting within cerebellar and motor pathways, maintaining dysfunction beyond the acute inflammatory response. The improvement seen with IVIG highlights the role of immune modulation and suggests that therapeutic interventions may indirectly reset aberrant epigenetic signaling, aiding recovery.

In this case, the patient's COVID-19 vaccination status is unknown; however, it remains an important factor in assessing post-infectious neurological syndromes. Brogna et al. recently demonstrated that using a proteomic mass spectrometry approach, recombinant Spike protein fragments encoded by mRNA vaccines can be detected in the blood of vaccinated individuals for up to 187 days after vaccination, regardless of antibody levels [5]. Importantly, these vaccine-specific “PP-Spike” fragments were not found in unvaccinated individuals, including those previously infected with SARS-CoV-2. These findings highlight the potential of proteomic tests to clarify vaccination history when serologic data are unclear and suggest that the persistence of recombinant Spike may influence the immune environment involved in post-viral neurological issues. In our case, the absence of vaccination data limits interpretation, but future research including vaccination details and direct proteomic analysis could provide critical insights into the mechanisms involved.

Emerging evidence also links the gut-brain axis to COVID-19-related effects. Toxin-like peptides similar to conotoxins, along with phospholipases, phosphodiesterases, zinc-metalloproteinases, and bradykinins, have been detected in the blood, feces, and urine of COVID-19 patients. Additionally, spontaneous SARS-CoV-2 replication has been observed in bacterial cultures of feces for 30 days or longer, indicating that parts of the gut microbiome might serve as reservoirs or influence viral persistence [6]. These findings suggest that COVID-19-related neurological syndromes could partly result from systemic effects of microbiota-derived peptides or ongoing viral-bacterial interactions that exacerbate neuroinflammation and disrupt neuronal signaling. In our patient with post-infectious myoclonus-ataxia, this data highlights the importance of considering host-microbiome-virus interactions as potential factors in disease development and as targets for future research.

Although the patient was insured, she faced significant barriers to accessing proper healthcare due to repeated denials of IVIG treatment by her insurance provider. This case highlights systemic flaws in the healthcare system, where having insurance coverage alone does not ensure adequate care. It emphasizes the broader impact of external factors such as policy restrictions, financial constraints, and administrative gatekeeping, which can directly affect patient outcomes [7]. Over the three years she went without the recommended treatment, her physical and mental health worsened, illustrating the toll of inadequate insurance practices. Furthermore, it remains uncertain whether the delay in receiving IVIG will cause long-term neurological damage. The full consequences of this lapse in care may not be evident for years, raising serious concerns about the lasting effects of delayed intervention in complex post-viral cases.

A formal ophthalmological assessment was not available at the time of evaluation; however, observing intermittent right-sided nystagmus during multiple clinical visits suggests possible involvement of the vestibulo-ocular pathway. Although the patient did not exhibit the full clinical features of opsoclonus-myoclonus syndrome (OMS), which usually include chaotic, multidirectional eye movements and more significant ocular motor instability, the isolated nystagmus warrants consideration within the broader range of post-COVID-19 immune-mediated neurological disorders [8]. Several published cases describe OMS or related ocular motor issues following SARS-CoV-2 infection, supporting the idea of partial or developing symptoms. In this case, the nystagmus might indicate a mild or incomplete form of brainstem or cerebellar dysfunction within the same immune-mediated spectrum.

Pathophysiology

The etiology of post-COVID-19 myoclonus and ataxia is not fully understood; nevertheless, converging data suggest an immune-mediated, para-infectious, or post-infectious process rather than direct viral invasion of the nervous system. The occurrence of delayed neurological symptoms after the clearance of acute SARS-CoV-2 infection, along with symptom improvement after treatment, strongly suggests autoimmune dysfunction and neuroinflammatory pathways.

One well-known theory compares it to opsoclonus-myoclonus-ataxia syndrome (OMAS), a neurological disorder that is usually brought on by post-infectious or paraneoplastic autoimmunity. Autoantibodies in OMAS impair neuronal inhibition in the cerebellum, leading to disinhibition of the deep cerebellar nuclei and subsequent hyperexcitability of the cortical motor and oculomotor regions. These autoantibodies often target Purkinje cells or cerebellar structures [9]. Clinical symptoms, such as ataxia, myoclonus, and ocular motor dysfunction, including nystagmus or opsoclonus, are all influenced by this disinhibition. One case showed cerebellar and striatal elevated metabolism on fluorodeoxyglucose positron emission tomography (FDG-PET) imaging, as well as CSF and serum antibodies targeting Purkinje cells, hippocampus neurons, and striatal neurons, despite the findings of autoantibody panels in COVID-19-associated cases being frequently negative10. These findings are consistent with immune-mediated cerebellar dysfunction.

Furthermore, the cerebellum may be a crucial part in the development of post-infectious ataxia and is often linked to autoimmune neurological diseases. The importance of active immune-related inflammation, rather than structural degradation, is further supported by hypermetabolism in the cerebellum, which has been previously reported in OMAS and paraneoplastic cerebellar degeneration [10]. It may also contribute to the development of cortical-subcortical myoclonus through abnormal cerebellar output to the motor cortex [11].

**Figure 2:**
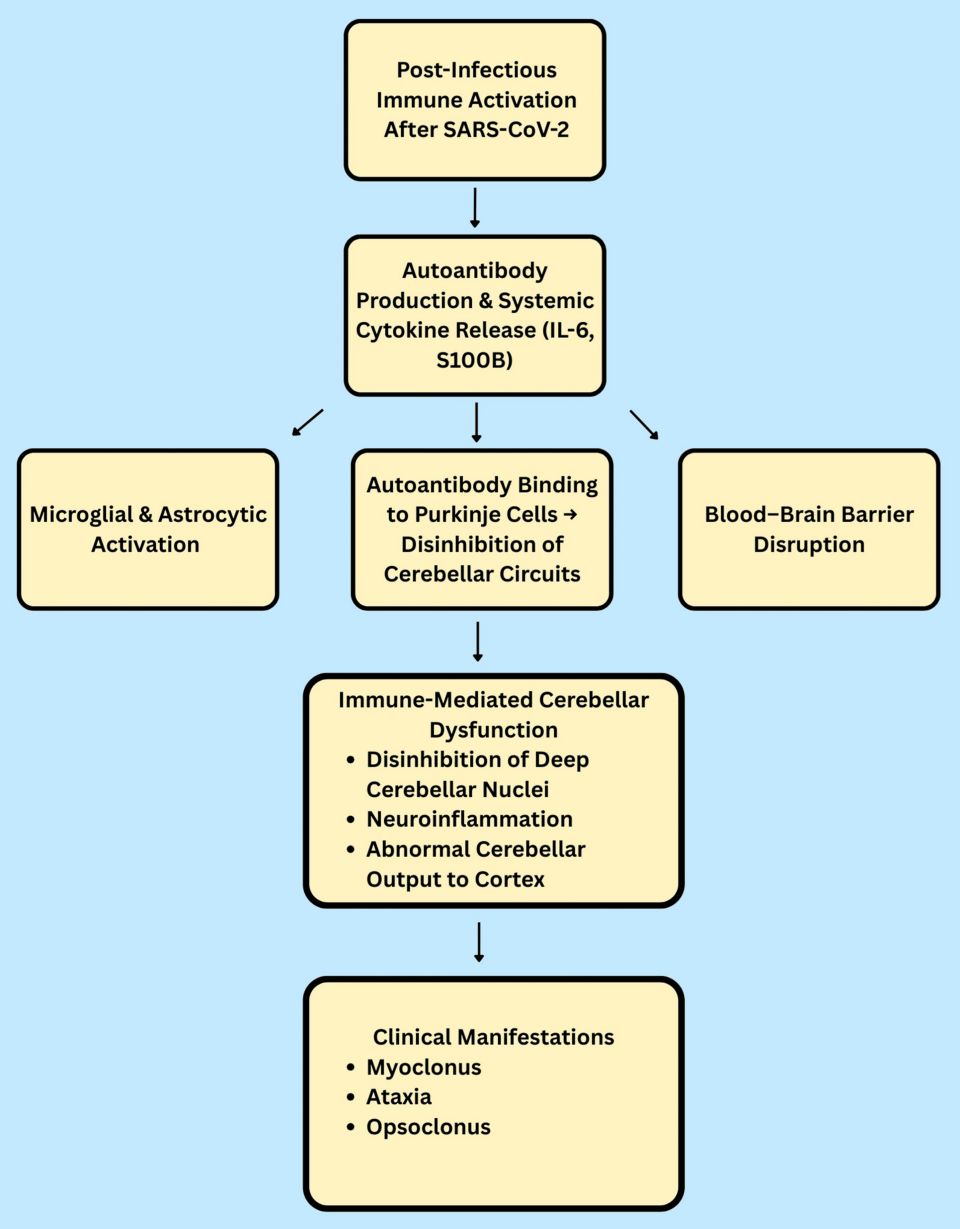
Proposed pathophysiological mechanism of post-COVID myoclonus-ataxia syndrome. Key takeaway: Immune-mediated cerebellar and brainstem dysfunction may underlie symptoms, consistent with improvement following IVIG therapy. IL-6: interleukin-6; TNF-α: tumor necrosis factor-alpha; BBB: blood-brain barrier; IVIG: intravenous immunoglobulin.

Besides autoimmunity mediated by antibodies, a para-infectious inflammatory response could also be key. Elevated serum or CSF levels of interleukin-6 (IL-6) and S100B, a marker of astroglial activation and blood-brain barrier (BBB) disruption, were observed in several cases of post-COVID myoclonus and ataxia [10-12]. In vulnerable areas of the brain, such as the cerebellum and brainstem, neuroinflammation caused by systemic cytokine production may activate microglia and astrocytes, leading to secondary neuronal dysfunction. The proposed mechanisms are illustrated in Figure 2. 

Together, these findings suggest that post-COVID neurological issues may stem from multiple overlapping mechanisms, including changes in cortical excitability, disruption of the gut-brain axis, and immune-related cerebellar dysfunction.

Limitations

The patient's vaccination status was unknown, which limits the ability to distinguish whether immune priming from prior vaccination contributed to the inflammatory response. Additionally, although the presentation was consistent with post-infectious immune-mediated ocular motor dysfunction, isolated nystagmus alone does not meet full criteria for opsoclonus-myoclonus syndrome, and not all ancillary evaluations (e.g., detailed ocular motor recording, expanded paraneoplastic panel) were performed. While alternative causes, such as structural disease, metabolic derangements, and medication effects, were clinically excluded, the absence of exhaustive testing warrants caution in drawing definitive mechanistic conclusions. These factors should be considered when interpreting the case, though the patient’s clinical course and improvement following IVIG remain consistent with reported post-COVID immune-mediated neurologic syndromes.

This report is limited by its single-patient design, which reduces the generalizability of the findings. Although the timing of the SARS-CoV-2 infection strongly suggests a post-infectious etiology, causality between the viral illness and subsequent neurological manifestations cannot be definitively proven. The lack of cerebrospinal fluid analysis and neuronal antibody testing further restricts the immunological understanding of the patient's condition. Furthermore, the prolonged delay in initiating IVIG therapy, largely due to repeated insurance denials, complicates the assessment of treatment effectiveness, as it is unclear whether earlier intervention could have changed the disease course or long-term outlook. Future studies involving larger patient cohorts, standardized immunological workups, and timely therapeutic interventions will be essential to confirm these findings and improve treatment approaches for post-COVID neurological syndromes.

Future implications

A substantial amount of follow-up will be needed to fully understand the long-term effects of neurological complications after COVID-19 infection. Given that the chronic effects of the respiratory illness itself are still not completely elucidated, the long-term neurological outcomes over time remain even more uncertain. We are currently in an observational phase, monitoring how post-COVID neurological symptoms, such as myoclonus and ataxia, manifest across patients. However, predicting the future health paths of these individuals remains challenging. Even after clinical symptoms disappear, concern remains that underlying neuronal damage, immune system imbalance, or subtle cognitive issues may persist. Additionally, patients might face a higher risk of recurrence or developing neurodegenerative diseases due to ongoing or abnormal immune responses. Continuous monitoring and longitudinal studies will be essential to evaluate these potential outcomes, guide ongoing care, and develop strategies to mitigate future risks in affected individuals.

## Conclusions

This case highlights a rare presentation of post-COVID myoclonus-ataxia syndrome in an immunocompetent adult, adding to the growing range of neurological complications following SARS-CoV-2 infection. The patient's limited response to corticosteroid therapy, followed by rapid and significant improvement with intravenous immunoglobulin (IVIG), emphasizes the need to consider antibody-mediated mechanisms when initial anti-inflammatory treatments do not work. Notably, this case also shows how insurance-related barriers and delayed access to proper care can lead to extended neurological issues and a decline in quality of life. By integrating clinical, therapeutic, and systemic insights, this report provides valuable lessons for the timely identification and management of post-infectious neuroimmune syndromes.
